# Contact neuro-endoscopy-assisted cerebral hematoma evacuation under direct vision

**DOI:** 10.3389/fsurg.2024.1351291

**Published:** 2024-03-07

**Authors:** Na Lu, Dong Qiao, ChengJiang Xue, YeGuang Pang

**Affiliations:** ^1^ Department of Neurosurgery, Qingdao Huangdao District Central Hospital, Qingdao, China; ^2^ Department of Clinical Medicine, Binzhou Medical University, Binzhou, China

**Keywords:** contact endoscopy, portable endoscopy, endoscopy, intracerebral hemorrhage, comprehensive direct visualization

## Abstract

Neuro-endoscopic hematoma evacuation is a crucial therapeutic approach for intracerebral hemorrhage. Our research team has developed a portable and contact neuro-endoscopy technique to enhance the conventional endoscopy procedure. compared to traditional endoscopy, this innovative approach involves miniaturizing the lens, light source, and camera system. These components are integrated into a stainless steel tube with a diameter of 4 mm, referred to as the portable endoscopy in this study. The portable endoscopy is powered by a USB cable and the video is displayed on a tablet computer. This portable endoscope facilitates easier operation with both hands by a single surgeon.

## 1 Introduction

Intracerebral hemorrhage (ICH) affects >1 million people annually worldwide and is the deadliest and most disabling type of stroke (1, 2). Hematoma removal surgery serves as an effective approach to alleviate hematoma-induced pressure, reduce intracranial pressure, and mitigate secondary injury, thereby representing a viable treatment option for cerebral hemorrhage (3, 4). Commonly employed surgical techniques encompass microscopic hematoma removal, neuro-endoscopic hematoma removal, puncture, and catheter hematoma drainage (5, 6). The selection of the surgical approach is contingent upon the hematoma's specific characteristics, necessitating a tailored approach (7). Based on a comprehensive analysis encompassing a total of 3,603 patients (8), it was determined that the utilization of neuro-endoscopy-assisted techniques for hematoma removal emerges as a highly effective intervention, exhibiting the capacity to enhance patients' functional prognosis and mitigate mortality rates. This can be attributed to the procedure's minimal invasiveness and prompt elimination of the hematoma.

Two primary phases are involved in the process of neuro-endoscopic hematoma removal, namely the establishment of working channels and the subsequent removal of the hematoma. Previously, the direct visualization of the neuro endoscope and subsequent hematoma removal was only possible after the successful establishment of the working channel. The method employed for establishing the working channel, known as Endoport, involves the utilization of a brain puncture needle/working channel based on anatomical indicators on the body surface or under the guidance of neural navigation. Following the arrival of the hematoma, syringe suction is employed to ascertain the presence of the hematoma within the cavity.

One crucial approach to attaining precise cerebral hemorrhage surgery involves commencing from clinical requirements, integrating novel technologies, and consistently enhancing surgical instruments, thereby diminishing surgical complexity, minimizing surgical harm, and enhancing surgical outcomes (6). In the context of endoscopic hematoma removal, prior investigations primarily concentrated on enhancing puncture accuracy during the establishment of working channels through the utilization of neural navigation (9–12), robotic arm (13), and B-ultrasound-assisted positioning (14), The utilization of techniques such as image fusion and 3D model-assisted positioning (15) has been employed to facilitate the accurate guidance of puncture direction and depth, as well as to enable the precision of the puncture process. However, these methods are limited in their ability to provide real-time observation of the puncture needle head's interaction with brain tissue and eliminate the need for suction confirmation, thus preventing the achievement of direct visual puncture. During the hematoma removal phase, certain scholars endeavored to incorporate a lightweight and portable endoscope (16) along with the utilization of the “chopstick technique” (17) to enhance the flexibility and convenience of the operation within the working channel.

This study introduces a new minimally invasive endoscopic removal technique for intracerebral hemorrhage, which utilizes a novel contact endoscope to assist surgeons in locating the hematoma under direct vision. This technique allows for the avoidance of puncturing the blood vessel and minimizes the risk of deviation in the puncture position. By having direct visibility, surgeons can make slight adjustments to the puncture direction during the operation to ensure precise positioning. Once the puncture reaches the lower boundary of the hematoma, which is typically around 1 cm–2 cm, Consistent with the direction of contact endoscopic puncture, implant the endoscopic working channel independently developed by our team. (patent No. ZL201910865237.9) independently developed by our team alongside the contact endoscope, ensuring a more accurate positioning of the working channel. Then, the lens sheath of the contact endoscope is removed, transforming it into a portable endoscope. This portable endoscope is equipped with a miniature camera at the front end, serving as a light source similar to the traditional neuroendoscope. However, it is significantly lighter, weighing only 10 g, and it can clearly project the situation in the operating field onto the display screen, providing the surgeon with a very comfortable experience. Finally, the hematoma is suctioned and removed to complete the operation. For traditional endoscopic intracerebral hemorrhage surgery, this surgical method is an optimization and upgrade. What should be proposed here is that during the stage of finding and confirming the location of the hematoma, and inserting the endoscopic working channel,the novel portable and contact neuro-endoscope, capable of performing hematoma puncture with direct visualization while avoiding critical blood vessels and avoiding the need for suction confirmation. It should be noted here that any blind suction confirmation behavior, even under the guidance of navigation, may induce intracranial rebleeding or brain damage. Furthermore, it enables complete endoscopic hematoma removal under direct visualization, thereby minimizing collateral damage caused by the puncture. The article primarily focuses on elucidating the composition of portable and contact neuro-endoscopy. It provides insights into the operational procedure and clinical experience through the analysis of relevant cases.

## 2 Materials and equipment

### 2.1 Patient selection

The study group consisted of 30 consecutive adult patients with supratentorial HICH treated between June 2020 and June 2023. The inclusion criteria were spontaneous supratentorial HICH confirmed on brain CT scans with hematoma volume > 30 ml, admission to hospital within 24 h of ictus, and adult age patients with a Glasgow Coma Scale (GCS) score ≥ 5. The patients underwent CT angiography to exclude underlying structural vascular disease. The exclusion criteria were hemorrhage caused by tumor, trauma, coagulopathy, aneurysm, arteriovenous malformation, hemorrhage after infarction, and use of antiplatelet or anticoagulant drugs over a long period of time. The study was approved by the Chinese Qingdao Huangdao District Central Hospital medical ethics committee. This article chooses one of them to illustrate this new surgical method.

### 2.2 Equipment

Disposable tissue retractor expansion catheter(patent No. ZL201910865237.9, ZL201910867098.3, ZL201921535779.1. Medical device registration number 20212021648. KZ-III type 13070), Made in Changsha Kezhong Medical Technology Co., LTD, China. It consists of three parts: an endoscopic working channel, a contact endoscope and a portable endoscope, camera system.

### 2.3 The fundamental principles and structural composition of a portable and contact endoscopic system

The contact endoscope (18), is an extension of the traditional endoscope, by making contact with the object, the rod mirror effectively establishes a fixed distance (known as the object distance) between the object and the convex lens, ensuring that the contact object remains consistently within the clear imaging interval. Notably, the curved lens end of the rod mirror serves to prevent any harm to brain tissue, while the flat tail end directly connects with the endoscopic lens, The contact endoscope retains the ability to capture clear images within brain tissue or blood during the puncture procedure.

The imaging principle of the portable endoscope (16) aligns with that of the conventional endoscope. The primary enhancement lies in the reduction of size for the optical imaging system, cold light source, and electronic camera system, which are integrated into the front end of a 4 mm stainless steel tube. Additionally, the portable endoscope eliminates the need for assembly during usage and eliminates operations such as focusing and white balance. Moreover, portable endoscopes employ lighter lenses. Simultaneously, the imaging system incorporates a low-power Complementary Metal Oxide Semiconductor (CMOS) sensor, thereby enabling the endoscope to possess a mere weight of 6 g. Additionally, this system facilitates the utilization of a USB cable, an ordinary tablet computer power supply, and a display, rendering it a plug-and-play device that is both lightweight and convenient. To enable direct visualization during the crucial stages of hematoma puncture and removal, it is necessary to convert the functionality of endoscopes from portable to contact endoscopes (18). In this research, a portable endoscope was modified by attaching an endoscopic sheath and incorporating a rod mirror at the distal end of the outer sheath.

**Figure 1:**
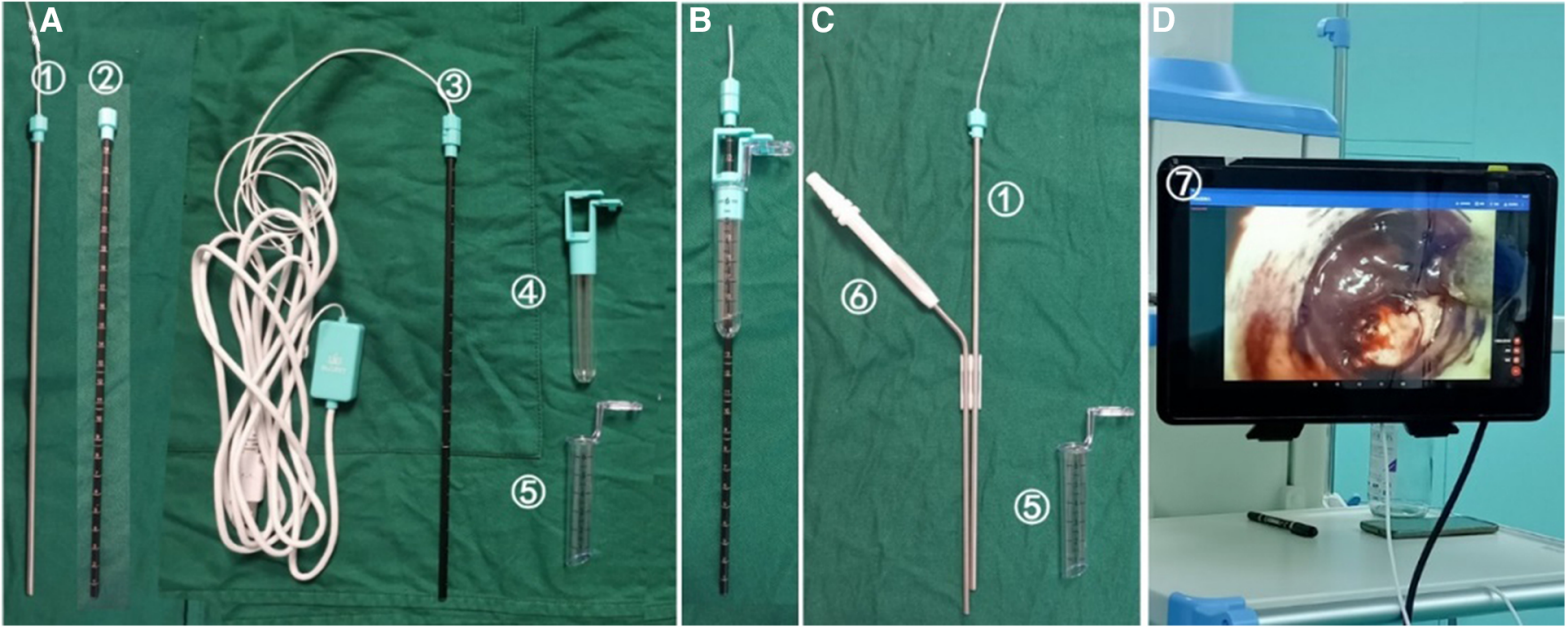
Composition of portable and contact endoscopic system(KZ-III type 13070, Disposable tissue retractor expansion catheter, Made in Changsha Kezhong Medical Technology Co., LTD, China). (**A**) The portable endoscope (①) and the outer sheath of the endoscope (②) form a contact endoscope (③), which can puncture the brain tissue under direct vision. (**B**) After arriving at the hematoma, push the working channel (⑤) and the working channel tube core (④) into the hematoma to establish the working channel. (**C**) The tube core of the working channel and the outer sheath of the endoscope were extracted, and the working channel was indurated in the brain (⑤). The portable endoscope (①) and aspirator (⑥) were used to remove the hematoma under direct vision. (**D**) The image is connected to the display via USB ⑦.

In conjunction with the aforementioned portable endoscope, endoscope sheath, and USB cable, surgical instruments encompass a working channel, a working channel tube core, and a display system. Please refer to Figure 1 for further elaboration. The portable endoscope, measuring 4 mm in diameter, functions as a 0° endoscope when inserted into the outer sheath, thereby forming a contact endoscope. The working channel is available in various specifications, such as a diameter of 13 mm and a length of 70 mm, as well as a diameter of 16 mm and a length of 90 mm, among others. Due to its slender design, the portable endoscope can be effectively integrated with a specialized aspirator, enabling the concurrent operation of both instruments with a single hand. During puncture, the portable endoscope was inserted into the outer sheath of the endoscope and assembled into a contact endoscope to achieve puncture under direct vision. After the working channel is established, the portable endoscope is removed and can be used like a traditional endoscope for hematoma removal under direct vision.

## 3 Methods

### 3.1 operation process and skills of contact endoscopic system

#### 3.1.1 Device preparation

① The contact endoscope should be assembled by inserting the portable endoscope into the outer sheath of the endoscope, rotating the end of the endoscope, and securing it (Figure 1A). ② The display should be connected by inserting the USB cable into the tablet computer, activating the endoscopic light source, and displaying the image (Figure 1D). ③ The working channel should be connected by locking and securing the working channel and the working channel tube core. The contact endoscope should be passed through the center of the tube core and positioned at the end of the contact endoscope to secure the contact endoscope and the working channel (Figure 1B).

#### 3.1.2 Direct view puncture

**Figure 2:**
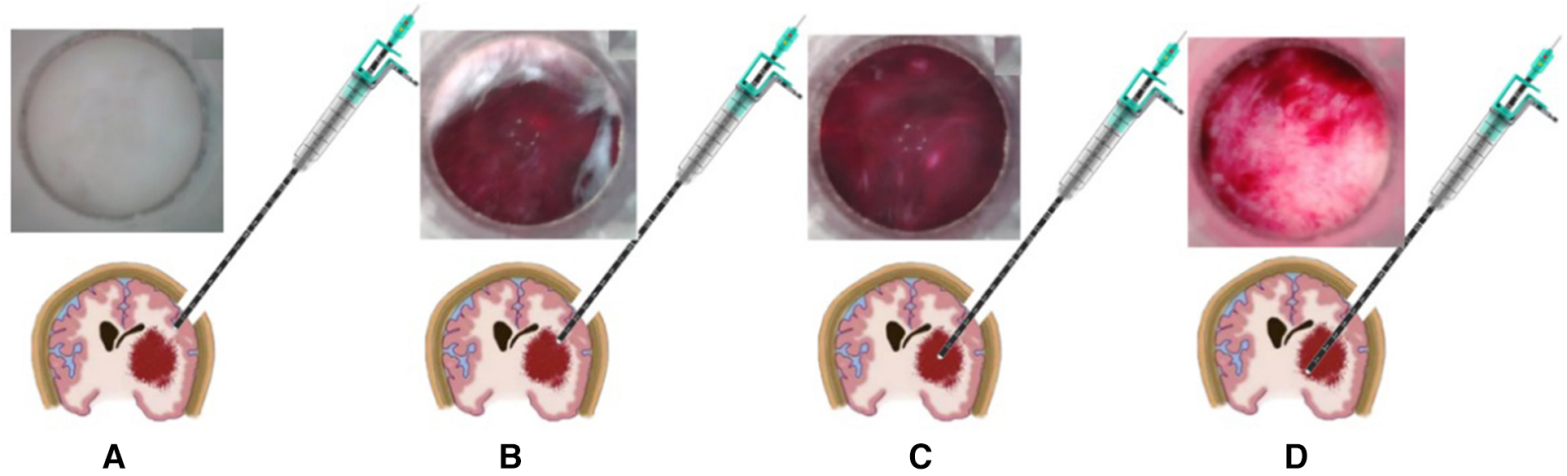
The schematic diagram depicting the process of puncture under direct view. (**A**) The placement of the contact endoscope within the brain tissue. (**B**) The puncture reaching the upper boundary of the hematoma, (**C**) the contact endoscope within the hematoma. (**D**) The contact endoscope investigating the base of the hematoma.

In the process of contact endoscopic puncture, a gradual puncture is performed towards the central region of the hematoma, following the predetermined path established during preoperative planning. Real-time visualization on the display screen allows for the observation of brain tissue displacement and blunt separation. Caution is exercised to prevent inadvertent damage to prominent blood vessels located beneath the cortex, while ensuring that the hematoma remains within the field of vision, indicating a successful puncture. Once the contact endoscope is inserted into the hematoma cavity, adjustments are made to determine the depth and angle in order to locate the base of the hematoma. For further details, refer to Figure 2.

To measure the length of a hematoma, utilize the scale present on the outer sheath of the endoscope. Upon reaching the upper edge of the hematoma with the contact inner lens end (Figure 2B), document the measurements of the outer sheath scale and the bone flap surface (referred to as scale 1). Proceed with the puncture until the contact inner lens end reaches the bottom of the hematoma (Figure 2D), and record the measurements of the outer sheath scale and the bone flap surface (referred to as scale 2), as well as the difference between scale 2 and scale 1. This difference represents the length of the hematoma in the direction of the puncture.

**Figure 3:**
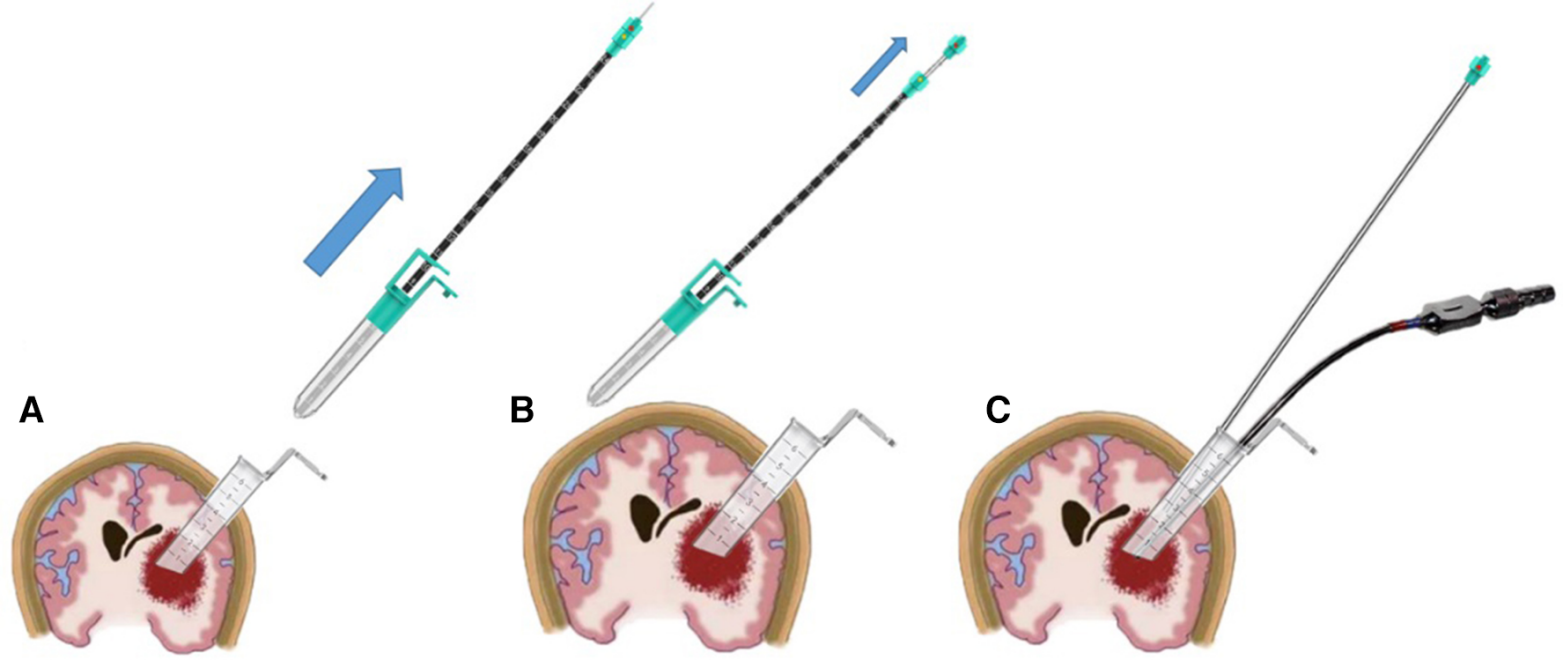
Illustrates a schematic diagram depicting the process of creating a functional channel. Firstly, the working channel tube core is unlocked and extracted from the working channel (**A**). Subsequently, the indenture working channel is positioned, and the contact endoscope's tail is rotated to detach it from the outer sheath of the endoscope, allowing for the extraction of the portable endoscope (**B**). Finally, the portable endoscope and aspirator are both positioned within a transparent working channel (**C**).

Create a functional pathway by manipulating the positioning of the contact endoscope, identifying the appropriate location for the working channel, disengaging the contact endoscope and the tube core of the working channel, and advancing the working channel and its tube core towards the inner lens end of the contact. Monitor the scale line indicated by the outer sheath of the endoscope, which becomes visible at the termination of the working channel, and cease movement upon reaching the 11 cm mark (using 7 cm as a hypothetical length example, noting that the scale line will vary accordingly). Subsequently, unlock the tube core and the working channel. The tube core and contact endoscope were removed, and the working channel was subsequently inserted into the brain. Subsequently, the contact endoscope was disassembled into a portable endoscope and an outer sheath of the endoscope, with the portable endoscope being inserted into the working channel. This is illustrated in Figure 3.

#### 3.1.3 Removal of hematoma under direct vision

Once the working channel has been established (Figure 3C), the working channel can be secured using either the assistant or the snake-like arm. Subsequently, the intracranial hematoma can be extracted using a portable endoscope, employing a technique similar to traditional endoscopic hematoma removal. In cases where bipolar electrocoagulation is necessary, the portable endoscope and suction device can be conjoined and immobilized (Figure 5C) to facilitate hematoma removal and achieve hemostasis using both hands.

#### 3.1.4 Operation skills of contact endoscope

First, the determination of the diameter of the bone flap is based on the specifications of the working channel, typically requiring a diameter approximately 1 cm larger than that of the channel. The majority of bone flaps have a diameter ranging from 2.5 cm to 3 cm. Second, The contact endoscope consists of a portable endoscope and the outer sheath, which can be locked or unlocked as needed during the operation to accommodate different scenarios. Third, The outer sheath of the endoscope is equipped with a scale that allows for the measurement of hematoma length. This measurement can be combined with the reading obtained at the end of the working channel. The depth of placement of the working channel can be prevented by adhering to specific guidelines. Fourth, The dimensions of the working channel tube core placement vary depending on the specifications. In the case of a 7 cm length specification, the tail scale should be positioned 11 cm away from the outer sheath of the endoscope, while the front end of the working channel should be precisely 1 cm away from the bottom of the hematoma. Fifth, an oblique incision is present at the anterior aspect of the working channel. In the process of hematoma removal, the rotation of the working channel facilitates the extrusion of the hematoma, thereby reducing its displacement, and subsequently removing the hematoma by retreating. It is advisable to introduce the working channel along the longitudinal axis of the hematoma to the greatest extent possible while employing other surgical instruments within the working channel, with careful consideration given to gentle maneuvers.

### 3.2 Introduction of clinical cases

#### 3.2.1 Basic clinical situation

The 55-year-old male patient was admitted to the emergency department as a result of a sudden onset of consciousness disturbance that worsened over a period of approximately three hours. The patient had a previous medical history of hypertension and irregular medication adherence. Upon admission, the patient exhibited a Glasgow Coma Scale (GCS) score of 6, bilateral pupil isogenic, and a blood pressure reading of 173/89 mmHg. Additional clinical findings included neck resistance, right limb hemiplegia (lack of response to stimuli and muscle contraction), hypotonia, the ability to flex the right limb with tingling sensations, weakened physiological reflexes, and absence of pathological reflexes. The head CT scan revealed a dense image in the left basal ganglia, indicating the possibility of a cerebral hemorrhage. The CTA did not detect any vascular malformations, and no evident abnormalities were observed. The diagnosis of hypertensive cerebral hemorrhage with temporal gyri hernia should be considered. To prepare for decompressive craniectomy, assisted by the contact and portable neuro-endoscopic removal of the intracranial hematoma was performed along with right ventricular external drainage.

#### 3.2.2 Preoperative planning

**Figure 4:**
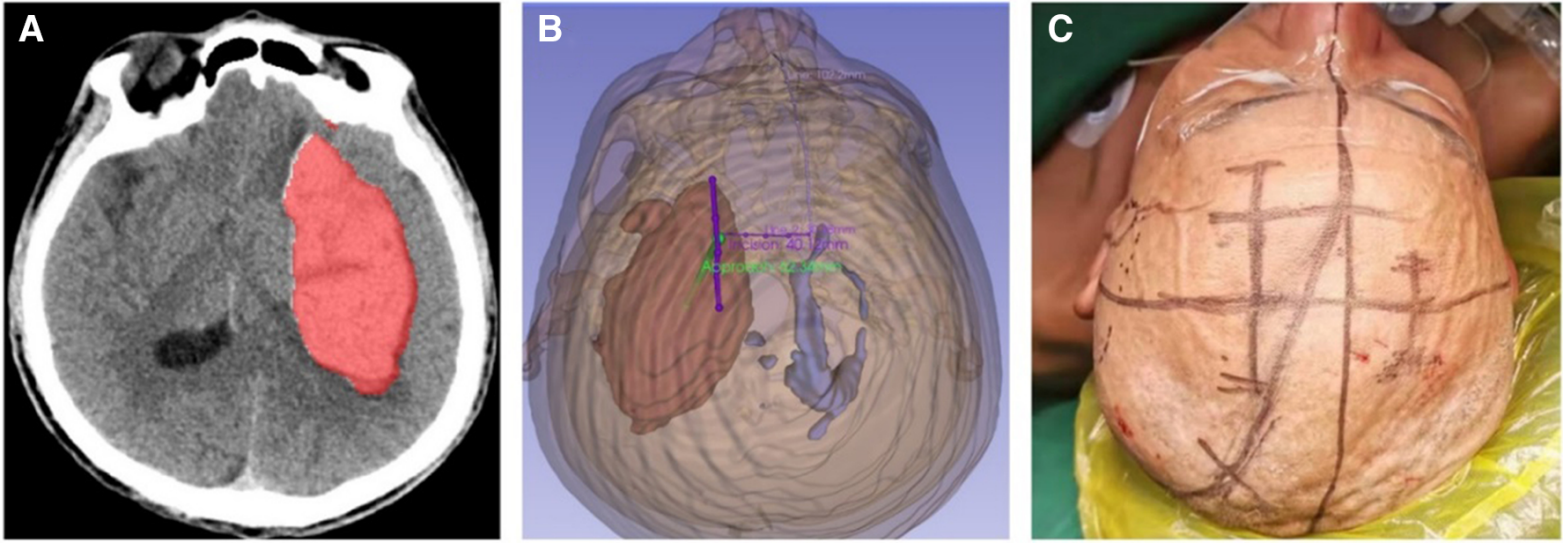
The automated quantitative tool utilized for cerebral hemorrhage acquires the segmentation file (**A**), which is then utilized to reconstruct the model in the 3D-Slicer software. Subsequently, the surgical plan is formulated (**B**), where the green color represents the surgical approach and the purple color represents the incision. The incisions are accurately traced on the patient's body surface in accordance with the preoperative plan (**C**).

The automatic quantitative tool for intracerebral hemorrhage (19) was utilized to segment key structures, including the hematoma, skull, and skin. The volume of the hematoma was measured to be 112.3 ml. Subsequently, the model was reconstructed using 3D-Slicer software (20), and a surgical plan was devised. The left hematoma was successfully cleared, and a right ventricular drainage procedure was performed. The mid-frontal approach was employed to remove the hematoma. A straight incision, approximately 5 cm in length, was made longitudinally, 2 cm–3 cm anterior to the coronal suture, with the center line opened 3 cm towards the left side. The puncture direction was the center of the hematoma (Figure 4).

#### 3.2.3 Surgical procedure

**Figure 5:**
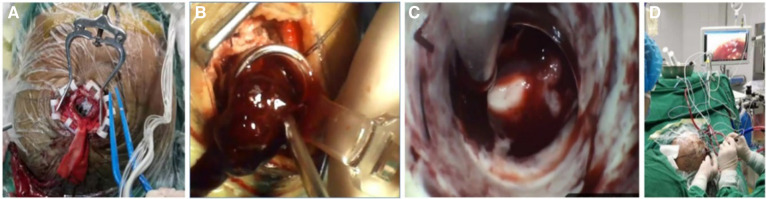
The surgical procedure is depicted. The incision measured approximately 5 cm, while the bone flap was approximately 2.5 cm in size (**A**). The establishment of the working channel facilitated the automatic outflow of the hematoma, thereby mitigating intracranial pressure (**B**). The utilization of a portable endoscope in conjunction with an aspirator to directly visualize and remove the hematoma. Finally (**C**), the simultaneous manual operation by a single individual to provide real-time observation on the screen (**D**).

The patient was positioned supine and administered general anesthesia for endotracheal intubation. Following the preoperative plan, a straight incision measuring 5 cm was made. A small bone flap with a diameter of approximately 2.5 cm–3 cm was created for the craniotomy. The contact neuro-endoscope was assembled and utilized for direct view puncture, confirming the presence of a hematoma and establishing a surgical channel. Subsequently, the contact endoscope was detached, the portable endoscope was extracted, and the hematoma was surgically removed under direct vision using a suction device and bipolar instrument. The procedure involves delicately manipulating the working channel to rotate its direction, systematically extracting hematoma in various orientations, while also employing flexible hemosic techniques such as fast yarn and fluid gelatin, and utilizing electric coagulation to identify the responsible blood vessels. The operation concludes once the working channel is gradually withdrawn to expose the hematoma, resulting in a notable reduction in brain tissue tension without any bleeding. Following the procedure, the postoperative plan is implemented in accordance with the preoperative strategy. the cotatntralateral intraventricular drainage tube with an intracranial pressure monitoring probe was inserted. The operation lasted about 80 min with little bleeding (Figure 5).

#### 3.2.4 Surgical effect and outcome

**Figure 6:**
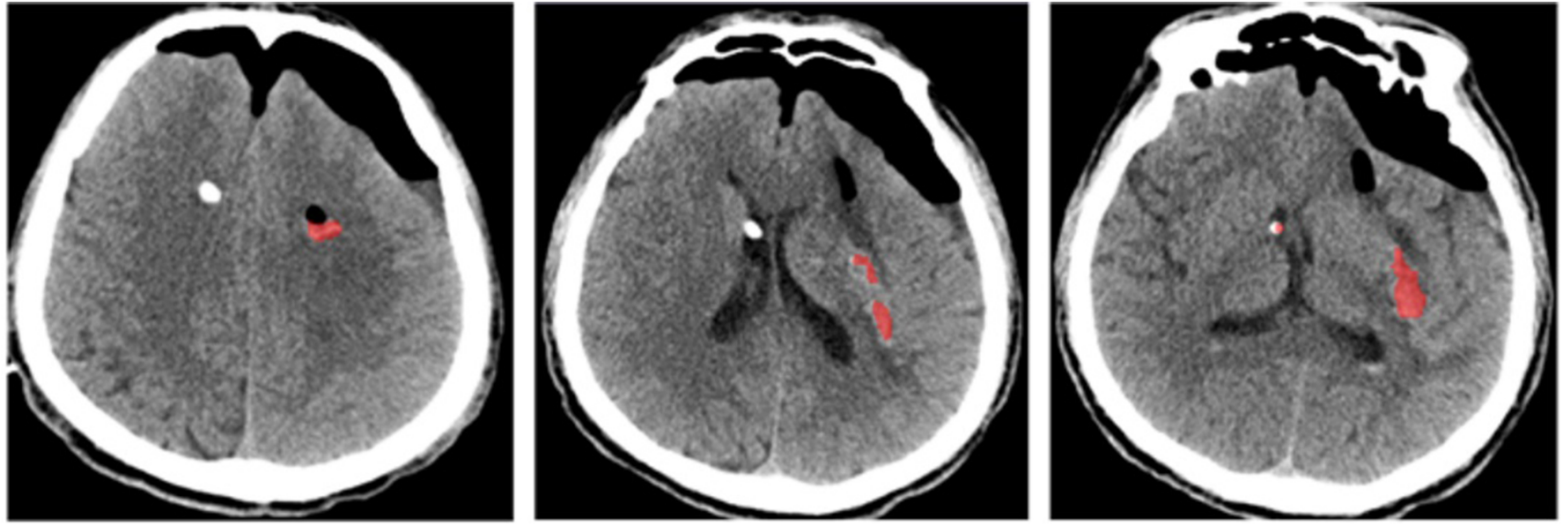
Postoperative review. Head CT examination 12 h after operation showed residual blood volume of 5.3 ml and hematoma clearance rate of 95.28%.

Twelve hours post-operation, a re-evaluation of the cranial CT revealed a residual blood volume of 5.3 ml, accompanied by a hematoma clearance rate of 95.28% (Figure 6). By the third day following the surgical procedure, the patient regained consciousness and achieved full wakefulness. Furthermore, at the 50-day mark post-surgery, the patient exhibited a perfect ADL score of 100, indicating complete independence in activities of daily living, including autonomous eating and movement.

## 4 Results and discussion

Neuro-endoscopic hematoma removal demonstrates the potential to achieve a balance between surgical efficacy and collateral damage (21). Several clinical trials and studies utilizing endoscopic visual technology for intracranial hematoma aspiration have been conducted both domestically and internationally, demonstrating positive clinical outcomes. “A Single Arm, Feasibility Study of Minimally Invasive Endoscopic Surgical Treatment with Apollo/Artemis for Supratentorial Intracerebral Hemorrhage (ICH) “is a prospective, multicenter single arm study. The primary objective is to provide an assessment of enrollment and follow up feasibility for this patient population being treated with the Apollo Minimally Invasive Surgical Treatment (MIES), and good clinical results and clinical evidence have been obtained (22, 23). “Early Minimally Invasive Removal of Intracerebral Hemorrhage (ENRICH): Study protocol for a multi-centered two-arm randomized adaptive trial”is a multicenter, randomized, adaptive clinical trial comparing standard medical management to early (<24 h) surgical hematoma evacuation using minimally invasive parafascicular surgery (MIPS) in the treatment of acute spontaneous supratentorial intracerebral hemorrhage, and good clinical effect and RCT clinical evidence were obtained (24).

In contrast to microscopic hematoma removal, endoscopic surgery enables the treatment of larger intracranial lesions through smaller incisions. Additionally, the utilization of direct visual guidance during the operation effectively minimizes the traction and injury to brain tissue, thereby safeguarding vital structures. Consequently, the overall incidence of surgical side effects is reduced. Furthermore, compared to puncture and drainage techniques, endoscopic surgery offers the advantage of timely hematoma removal.

The novel endoscope employed in this study incorporates two key enhancements. One aspect to consider is the utilization of a contact endoscope, which involves the incorporation of an outer sheath into the conventional endoscope. This modification, where in the head end encompasses a rod mirror, facilitates the uninterrupted imaging of brain tissue. Consequently, the contact endoscope is capable of executing direct-view puncture operations, thereby circumventing potential interference with blood vessels along the puncture path. Additionally, this advancement diminishes the necessity for multiple suctioning steps to confirm the presence of hematoma, while also enabling the measurement of hematoma length. Conversely, the advent of portable endoscopy has been made possible through advancements in electronic endoscopy technology. This progress has resulted in the production of more slender and lightweight endoscopes. The portable endoscope in this study is only 6 g, which effectively reduces the burden of doctors holding the lens. Simultaneously, the utilization of a suction device can rectify the aforementioned issue. By employing the “chopstick technology,” the endoscope and suction device can be manipulated single-handedly, allowing the other hand to operate additional instruments. The portable endoscope exhibits a high level of integration, eliminating the necessity for an independent light source, white balance adjustments, and other procedures. It can be effortlessly connected to a display via USB, enabling immediate and convenient usage. The innovation lies in the fact that the contact endoscope can be directly inserted into brain tissue for imaging at a 0 mm object distance. It is capable of imaging brain tissue and hematoma clearly, while the traditional endoscope can only image in transparent gas or liquid and cannot image in soft tissues similar to brain tissue. Additionally, the lightweight and highly integrated endoscope system and cold light source system are relatively convenient to operate. The contact endoscope does not require manual cleaning of the lens during the puncture procedure. The curved front end of the lens makes direct contact with brain tissue for imaging, utilizing a blunt separation technique. As the tissue at the front end gradually shifts due to friction with the lens during puncture, it acts as a self-cleaning mechanism akin to wiping. This process prevents the accumulation of stains on the lens that could potentially contaminate it, and any existing stains are effectively removed during the piercing process. During the utilization of a portable endoscope, lens cleaning is typically unnecessary throughout the operation, although instances may arise where it becomes imperative to remove stains from the lens. Endoscopic surgery is conducted entirely within a transparent sheath, known as the working channel, which serves to shield the brain tis-sue and prevent contact or contamination of the lens by blood. The hematoma and other tissues within the surgical site are situated at aconsiderable distance from the lens, with the optimal working distance being 25 mm for optimal image quality. Furthermore, the front lens of the endoscopic system is equipped with an anti-fog and anti-fouling coating.These factors have the potential to reduce the duration of the surgical procedure and facilitate its execution.

The new endoscope boasts a resolution of 1,280*720 pixels (1 K), slightly inferior to the current high-end 4 K optical endoscope. However, it adequately fulfills the requirements for clinical applications in cerebral hemorrhage surgery, And the image quality is constantly being optimized Additionally, efforts have been made to integrate the endoscope with stereotactic and neural navigation systems, expanding its potential applications and enhancing the safety of surgical procedures.

However, it is important to note that current endoscopes possess certain limitations (25). Akin to conventional endoscopy, the operative area is constrained by the working channel, necessitating enhanced surgical proficiency on the part of the operator, particularly in managing active hemorrhaging.

## 5 Conclusion

In summary, the present study introduces a novel neuro-endoscope that amalgamates the merits of portable and contact endoscopes, enabling comprehensive direct visualization during hematoma removal procedures. This innovation offers enhanced convenience for medical practitioners and contributes to the precision and minimally invasive nature of cerebral hemorrhage surgery. Consequently, further investigation into the clinical significance of this neuro-endoscope is warranted in future research endeavors.

## Data Availability

The original contributions presented in the study are included in the article/Supplementary Material, further inquiries can be directed to the corresponding author.

## References

[1] Qureshi AI Tuhrim S Broderick JP Batjer HH Hondo H Hanley DF. Spontaneous intracerebral hemorrhage. N Engl J Med. (2001) 344:1450–60. 10.1056/NEJM200105103441907 11346811

[2] Flaherty ML Haverbusch M Sekar P Kissela B Kleindorfer D Moomaw CJ Long-term mortality after intracerebral hemorrhage. Neurology. (2006) 66:1182–6. 10.1212/01.wnl.0000208400.08722.7c 16636234

[3] Spiotta AM Fiorella D Vargas J Khalessi A Hoit D Arthur A Initial multicenter technical experience with the Apollo device for minimally invasive intracerebral hematoma evacuation. Neurosurgery. (2015) 11(Suppl 2):243–51. 10.1227/NEU.0000000000000698 25714520

[4] Przybylowski CJ Ding D Starke RM Crowley RW Liu KC. Endoport-assisted surgery for the management of spontaneous intracerebral hemorrhage. J Clin Neurosci. (2015) 22:1727–32. 10.1016/j.jocn.2015.05.015 26238692

[5] Gil-Garcia CA Flores-Alvarez E Cebrian-Garcia R Mendoza-Lopez AC Gonzalez-Hermosillo LM Garcia-Blanco MD Essential topics about the imaging diagnosis and treatment of hemorrhagic stroke: a comprehensive review of the 2022 AHA guidelines. Curr Probl Cardiol. (2022) 47(11):101328. 10.1016/j.cpcardiol.2022.101328 35870549

[6] Dastur CK Yu W. Current management of spontaneous intracerebral hemorrhage. Stroke Vasc Neurol. (2017) 2(1):21–9. 10.1136/svn-2016-000047 28959487 PMC5435209

[7] Shoamanesh A Patrice Lindsay M Castellucci LA Cayley A Crowther M Wit KD Canadian Stroke best practice recommendations: management of spontaneous intracerebral hemorrhage, 7th edition update 2020. Int J Stroke. (2020) 16(3):321–41. 10.1177/1747493020968424 33174815

[8] Guo G Pan C Guo W Bai S Nie H Feng Y Efficacy and safety of four interventions for spontaneous supratentorial intracerebral hemorrhage: a network meta-analysis. J Neurointerv Surg. (2020) 12(6):598–604. 10.1136/neurintsurg-2019-015362 31900351

[9] Uchida D Nakatogawa H Yamazoe T Inenaga C Tanaka T. Neuroendoscopic surgery with a combination of image detectable sheath, intraoperative computed tomography scan, and navigation system improves accuracy and safety in minimally invasive evacuation of intracerebral hematoma: technical note. World Neurosurg. (2020) 133:1–7. 10.1016/j.wneu.2019.09.058 31541759

[10] Waran V Vairavan N Sia SF. A new expandable cannula system for endoscopic evacuation of intraparenchymal hemorrhages. J Neurosurg. (2009) 111:1127–30. 10.3171/2009.4.JNS081506 19408977

[11] Sun GC Chen XL Hou YZ. Image-guided endoscopic surgery for spontaneous supratentorial intracerebral hematoma. J Neurosurg. (2017) 127(3):537–42. 10.3171/2016.7.JNS16932 27636179

[12] Zhao YN Chen XL. Endoscopic treatment of hypertensive intracerebral hemorrhage: a technical review. Chronic Dis Transl Med. (2016) 2(3):140–6. 10.1016/j.cdtm.2016.11.002 29063035 PMC5643758

[13] Wu S Wang H Wang J Hu F Jiang W Lei T Effect of robot-assisted neuroendoscopic hematoma evacuation combined intracranial pressure monitoring for the treatment of hypertensive intracerebral hemorrhage. Front Neurol. (2021) 12:722924. 10.3389/fneur.2021.722924 34925205 PMC8674426

[14] Chartrain AG Hom D Bederson JB Mocco J Kellner CP. Intracavitary ultrasound (ICARUS): a neuro-endoscopic adaptation of intravascular ultrasound for intracerebral hemorrhage evacuation. J Neurointerv Surg. (2018) 10(7):e16. 10.1136/neurintsurg-2017-013188.rep 29563209

[15] Li Y Cheng H Li Z Zhao H Wang J Wang P Jin T Clinical value of 3D-printed navigation technology combined with neuroendoscopy for intracerebral hemorrhage. Transl Stroke Res. (2021) 12(6):1035–44. 10.1007/s12975-021-00893-6 33492652

[16] Cepek J Wilson C Denstedt J Stern N Razvi H Bjazevic J Portable endoscopic simulator for urologic training: a face/content and construct validity study. J Endourol. (2022) 36(11):1495–501. 10.1089/end.2022.0180 35546282

[17] Li W Li Z Wei C Yang X Ji Y Liu H. Microscopic and endoscopic “chopstick” technique removal of choroid plexus papilloma in the third ventricle of an infant: a case report with systematic review of literature. Front Oncol. (2023) 13:1182261. 10.3389/fonc.2023.1182261 37434973 PMC10332163

[18] Davaris N Lux A Esmaeili N Illanes A Boese A Friebe M Evaluation of vascular patterns using contact endoscopy and narrow-band imaging (CE-NBI) for the diagnosis of vocal fold malignancy. Cancers (Basel). (2020) 12(1):248. 10.3390/cancers12010248 31968528 PMC7016896

[19] Peng C Yang L Yi W Yidan L Yanglingxi W Qingtao Z Application of fused reality holographic image and navigation technology in the puncture treatment of hypertensive intracerebral hemorrhage. Front Neurosci. (2022) 16:850179. 10.3389/fnins.2022.850179 35360174 PMC8963409

[20] Liao R Liu L Song B Wan X Wang S Xu J. 3D-slicer software-assisted neuroendoscopic surgery in the treatment of hypertensive cerebral hemorrhage. Comput Math Methods Med. (2022) 2022:7156598. 10.1155/2022/7156598. eCollection 2022. 35222690 PMC8881139

[21] Guo W Liu H Tan Z Zhang X Gao J Zhang L Comparison of endoscopic evacuation, stereotactic aspiration, and craniotomy for treatment of basal ganglia hemorrhage. J Neurointerv Surg. (2020) 12(1):55–61. 10.1136/neurintsurg-2019-014962 31300535 PMC6996102

[22] Kellner CP Chartrain AG Nistal DA Scaggiante J Hom D Ghatan S The stereotactic intracerebral hemorrhage underwater blood aspiration (SCUBA) technique for minimally invasive endoscopic intracerebral hemorrhage evacuation. J Neurointerv Surg. (2018) 10(8):771–6. 10.1136/neurintsurg-2017-013719 29572265 PMC6278654

[23] Kellner CP Song R Pan J Nistal DA Scaggiante J Chartrain AG Long-term functional outcome following minimally invasive endoscopic intracerebral hemorrhage evacuation. J Neurointerv Surg. (2020) 12(5):489–94. 10.1136/neurintsurg-2019-015528 31915207 PMC7231458

[24] Ratcliff JJ Hall AJ Porto E Saville BR Lewis RJ Allen JW Early minimally invasive removal of intracerebral hemorrhage (ENRICH): study protocol for a multi-centered two-arm randomized adaptive trial. Front Neurol. (2023) 14:1126958. 10.3389/fneur.2023.1126958 37006503 PMC10061000

[25] Cai Q Li Z Wang W Ji B Liu J Chen Z Hemorrhagic stroke treated by transcranial neuro-endoscopic approach. Sci Rep. (2021) 11(1):11890. 10.1038/s41598-021-90927-8 34088921 PMC8178359

